# Optimizing Anesthesia for Extensive Extraoral Fungating Lesions: Strategies and Considerations

**DOI:** 10.7759/cureus.62221

**Published:** 2024-06-12

**Authors:** Samarpan Patel, Sanjot Ninave, Shakti Sagar

**Affiliations:** 1 Anesthesiology, Jawaharlal Nehru Medical College, Datta Meghe Institute of Higher Education & Research, Wardha, IND; 2 Pathology, Jawaharlal Nehru Medical College, Datta Meghe Institute of Higher Education & Research, Wardha, IND

**Keywords:** pre-anesthetic checkup, tracheal intubation, facemask ventilation, difficult airway, oral cancer

## Abstract

A large extraoral fungus, frequently seen in late head and neck cancers, poses serious difficulties for the management of anesthesia and surgery. Essential factors include preoperative optimization, airway assessment, intraoperative monitoring, and postoperative care. Risk mitigation and outcome optimization strategies are discussed, including appropriate airway management and hemodynamic monitoring. Ideal patient outcomes in situations of extensive extraoral fungation can be attained by a complete plan that integrates surgical expertise and anesthetic care. This case discusses the successful anesthetic management of a 55-year-old man undergoing composite resection with segmental mandibulectomy, appropriate neck dissection, free fibular flap, and scalp flap for squamous cell carcinoma of the lower labial mucosa with significant extraoral fungation.

## Introduction

According to the American Society of Anesthesiologists, a challenging airway is when a conventionally trained anesthesiologist experiences difficulty with facemask ventilation of the upper airway, difficulty with tracheal intubation, or both [1]. The intended surgical procedure influences the plan to manage the difficult airway, the patient’s current status and vital signs, medical and surgical history, and the airway examination [2]. Oral cancer with a large extraoral fungating mass and intraoral extension establishing airway management is the main priority while dealing with oral cancer for an anesthetist. When treating oral cancer, surgical intervention is the first line of treatment [3]. However, during oromaxillary surgery, anesthesia issues mainly stem from a limited mouth opening and a narrower interincisor space. The sixth most common cancer globally is oral cancer [3]. It is linked to tobacco and gutka use and is the most common cancer among men in India [4]. Patients who have oral cancer treated with radiation are more likely to experience difficulties with limited mouth opening, neck extension, and motion. Given these variables, anesthesiologists’ expertise and judgment will undoubtedly lower morbidity and death. The phrases “free flap,” “island flap,” “free autologous tissue transfer,” and “microvascular free tissue transfer” describe the process of transferring tissue from one area of the body to another to repair an already existing cut. The term “free” describes the transfer of tissue from the original location (donor) to a new location (receiver), where artery and venous anastomosis establish circulation. Free flap transplants can replace intestine segments, lymph nodes, bone, muscle, nerve, skin, and subcutaneous tissue. Microvascular anastomosis, which Goldwyn and Krizek pioneered in the 1960s, allowed for free tissue transplantation in animal models [5,6]. In 1973, the first known effective free flap procedure in a clinical context was carried out by Daniel and Taylor, initiating the discovery [7]. Large extraoral fungal masses frequently present difficulties for anesthesiologists because they hinder the airway during intubation and mask ventilation. Thorough preoperative evaluation and planning for airway management enable one to anticipate challenges and navigate them easily.

## Case presentation

**Figure 1:**
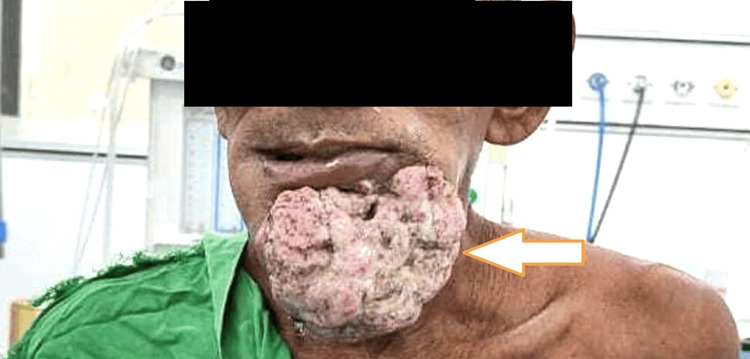
Image showing large extraoral fungation

An extraoral, huge fungating mass over the left chin region and a nonhealing ulcer over the left lower front of the jaw region were the reasons behind the 55-year-old male patient’s visit to the casualty ward in the last two years (Figure 1).

**Figure 2:**
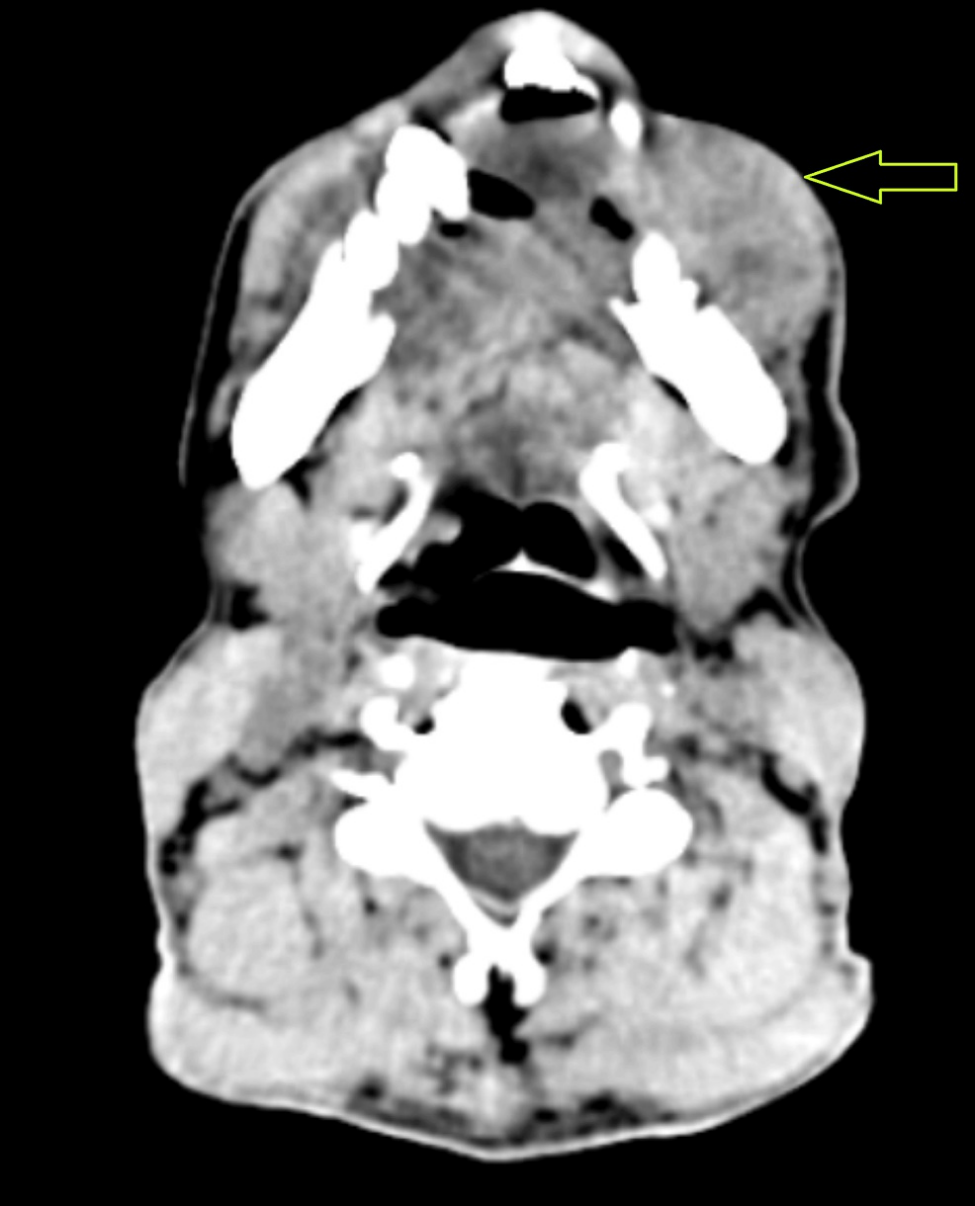
Contrast-enhanced CT showing extraoral fungating mass with no intraoral extension

After the initial evaluation, the patient was quickly referred to the oncological surgery section for additional testing. Upon detailed investigation, the individual disclosed a long history of alcohol addiction spanning three decades and tobacco usage lasting 25 years. The area of interest was promptly subjected to a biopsy, and the existence of squamous cell carcinoma was later confirmed by histological examination. A suitable neck dissection, free fibular flap, scalp flap, and segmental mandibulectomy were all planned as part of a composite resection. The patient had no surgical background or comorbidities upon examination before anesthesia. A mouth opening of two fingers and a Mallampati categorization of class IV, indicating limited visibility of the hard palate, were noted during the airway examination. There was restricted temporomandibular joint movement due to an accompanying painful mouth lesion, although neck movement seemed sufficient. All laboratory tests, including the complete blood count, kidney function test, and liver function test, revealed values within acceptable ranges. Contrast-enhanced CT of the neck showed an extraoral fungating mass with no intraoral extension, involving the parasymphysis region, mentalis, depressor labii inferioris, left depressor anguli oris, and left buccinators (Figure 2).

Except for the oral lesion, the patient’s overall condition was deemed appropriate for additional anesthetic examination and intervention. After comprehensive preoperative counseling and confirming nil by mouth, awake intubation with a fiberoptic bronchoscope was scheduled. The patient received IV fluids throughout the eight-hour fasting period. Upon moving to the operating table, two 18G catheters were placed on each hand after obtaining their consent for a high-risk course of treatment. The patient was topicalized with 4% lignocaine and oxygen for up to half an hour in the preoperative room before the procedure to prepare the airway down to the trachea. Additionally, 2% and 4% lignocaine were used for superior and recurrent laryngeal nerve blocks. Nasal drops containing xylometazoline were administered to both nostrils. A nasal airway, lubricated with lignocaine gel, was gradually inserted into the left nostril to aid in the placement of the endotracheal tube. The patient received 4 liters of oxygen for three minutes using a nasal prong for preoxygenation. After airway blocks preparation, intravenous injections (fentanyl 50 mcg, midazolam 1 mg, and glycopyrrolate 0.2 mg) were administered to induce drowsiness and analgesia. An 8-mm cuffed flexometallic tube was railroaded using a bronchoscope, carefully inserted into the left nostril, and guided to the carina level under direct view. Subsequently, intravenous injections of vecuronium (8 mg) and propofol (100 mg) were given. The patient was connected to a ventilator, and endotracheal tube securing was performed by surgeons using sutures once ventilation was confirmed by a 5-point auscultation and capnography. Ryles tube insertion and throat packing were then completed. The patient was maintained on sevoflurane as an inhalation agent, with a mixture of two liters of oxygen and two liters of nitrous oxide. Once the patient was prepared, surgeons proceeded with a lengthy 14-hour surgery, carefully managing fluids and monitoring everything with arterial blood gas.

Using an 8.0-mm cuffed tracheal tube, an intraoperative tracheostomy was performed. After the surgery, the patient was kept on a ventilator and sedated with vecuronium and midazolam infusions at a rate of 4 mg/hour each in the ICU. On the first postoperative day, infusions were stopped, and the patient was gradually weaned off the ventilator, transitioning to room air while regularly monitoring their oxygen saturation. Decannulation was performed on the seventh postoperative day to close the tracheostomy opening, and on day 12, the patient was discharged without incident.

## Discussion

The four main factors that are most commonly associated with the development of cancer include chewing tobacco and gutka, using pan masala, and having an unhealthy diet [8,9]. Head and neck cancers are among the top 10 most prevalent types of cancer worldwide [8]. The bulk of individuals with head and neck cancer exhibit delayed symptoms related to their therapy of the airways [10]. In certain situations, both intubation and extubation may be difficult. This is among the anesthetic concerns because it may give rise to further difficulties in the management of the airway during surgery.

Patients with head and neck cancer typically suffer upper airway distortion as well as soft tissue fixation and hardening brought on by radiation therapy. A preoperative evaluation should include a history of chemotherapy and radiation therapy. Due to the possibility of postprocedural tracheal stenosis, it is crucial to find out about any previous history of tracheostomy. Patients may experience decreased jaw opening or neck extension due to tissue fibrosis after radiation therapy. Keep track of the chemotherapy drugs utilized, the time and distance since the last cycle, and other pertinent information. Airway compromise from stenosis, tumors, nerve injury, or edema can be detected by symptoms including dyspnea, stridor, and a hoarse voice [11]. One technique to stratify the risk of difficult intubation would be to perform a thorough airway assessment using the criteria found in the L-E-M-O-N method. One can promptly evaluate for possibly challenging airways by applying the LEMON Law [12]. According to the criteria, a score of 10 can be assigned; higher scores are linked to difficult intubation and poor glottic visibility.

Anesthesia-related concerns include the following: if the patient has periglottic or perioral growth, bag and mask ventilation could be complicated; exophytic tumors can be easily broken up and removed from their distant locations with laryngoscopes. Due to their tendency to bleed readily, these tumors impede subsequent glottic visibility; if they spread undetected to the base of the tongue, tongue fixation ensues, complicating laryngoscopy and intubation. Patients may find it more challenging to undergo laryngoscopy and glottic visualization due to tumor invasion. Although video laryngoscopes offer better airway images, they require more space to insert and may be able to loosen the tumor. Radiation exposure in the past exacerbates the illness [13].

Because of its efficacy and safety profile, awake fiberoptic intubation is considered the most effective technique for managing troubling airways. However, the successful execution of this procedure necessitates the expertise of highly skilled anesthesiologists with specialized training. The meticulous navigation of the fiberoptic scope through the upper airway demands precision and finesse, underscoring the paramount importance of technical proficiency in this advanced airway management approach [14,15]. A sedative may make awake fiber optic intubation a little easier. For this reason, dexmedetomidine infusion [16] is quite effective. Airway blocks (superior laryngeal nerve, glossopharyngeal nerve, and recurrent laryngeal nerve blocks) and local anesthetic topicalization of the airway, either by lignocaine nebulization or the “spray as you go” approach, may make airway instrumentation simpler [17].

Each method has some possible drawbacks. Blind nasal intubation typically necessitates multiple tries with an endotracheal tube inserted via the nose, supported by the contralateral hand’s external probing of the glottis and the existence of the end-tidal carbon dioxide trace. Frequent efforts may result in hemorrhage, laryngeal edema, and airway loss in a young patient due to the possibility of being missed [18]. Retrograde intubations are challenging to execute and infrequently used since they require extensive airway management experience. Because tracheostomy is an invasive procedure with associated diseases and conditions, it is saved for critical scenarios [18].

**Figure 3:**
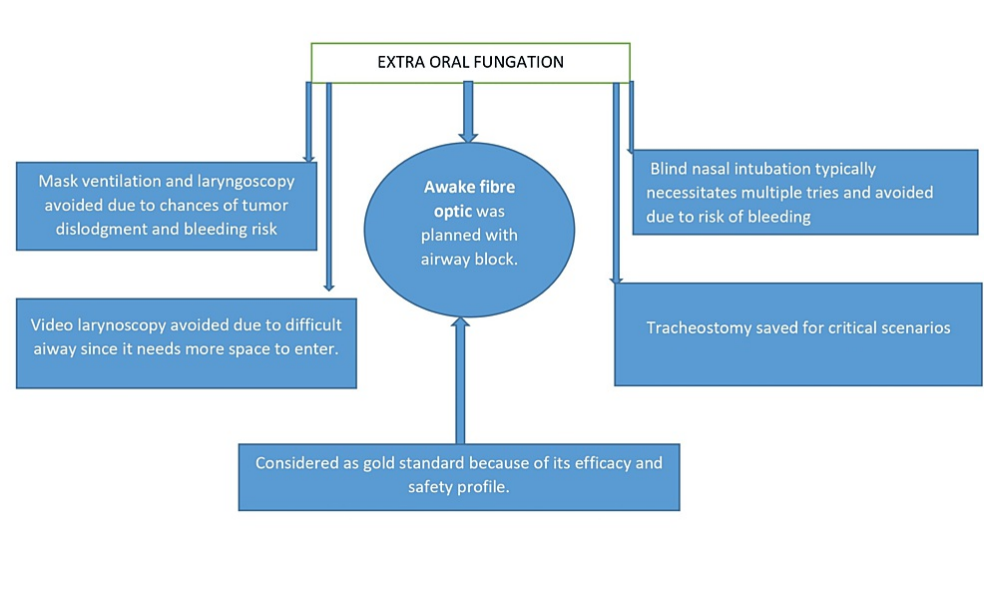
Consideration for managing difficult airway in extraoral fungation

Every airway management plan, such as awake fiber optic, video laryngoscopy, laryngoscopy, blind nasal intubation, and tracheostomy, has specific factors to be taken into account while selecting the optimal airway strategy (Figure 3).

**Figure 4:**
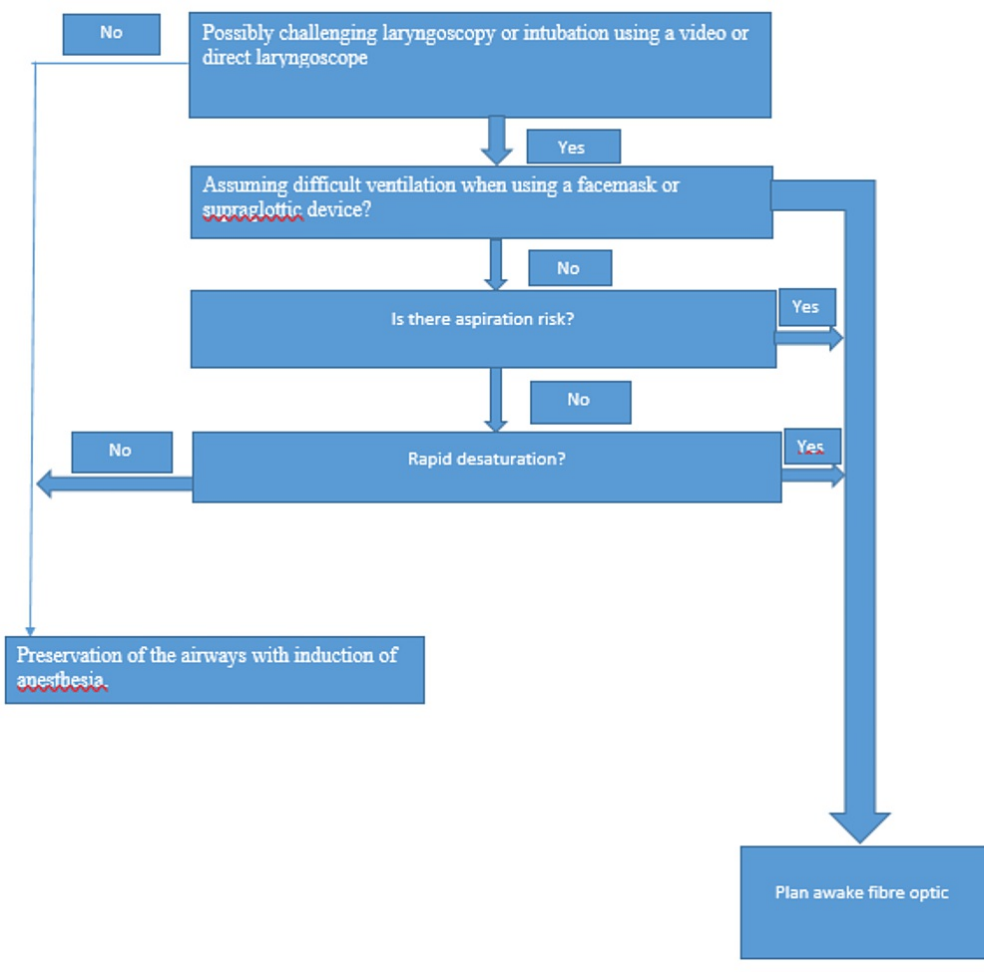
Strategies to secure the airway in extraoral fungation

Managing the airway in patients with extraoral fungation that involves neighboring structures is highly complex and demands thorough planning and strategic approaches (Figure 4).

## Conclusions

As science and technology have advanced, the fiberoptic bronchoscope has emerged as the benchmark for treating unexpectedly complicated airways that will likely require difficult ventilation. In this study, a difficult airway was anticipated, with the risk of dislodgment of exophytic tumors and bleeding; mask ventilation and laryngoscopy were avoided. Proper patient counseling regarding anesthesia technique and effective airway block is essential for smooth, awake fiberoptic intubation. Good postoperative outcomes, an early recovery, and hospital discharge are all attributed to appropriate intraoperative hemodynamic control and airway management.
